# The Prevalence of Physical Activity and Sedentary Behaviours Relative to Obesity among Adolescents from Al-Ahsa, Saudi Arabia: Rural versus Urban Variations

**DOI:** 10.1155/2012/417589

**Published:** 2012-01-23

**Authors:** Anwar A. Al-Nuaim, Yahya Al-Nakeeb, Mark Lyons, Hazzaa M. Al-Hazzaa, Alan Nevill, Peter Collins, Michael J. Duncan

**Affiliations:** ^1^School of Human Sciences, Newman University College, Genners Lane, Bartley Green, Birmingham B32 3NT, UK; ^2^Exercise Physiology Laboratory, King Saud University, P.O. Box 2458, Riyadh 11451, Saudi Arabia; ^3^School of Performing Arts and Leisure, University of Wolverhampton, Walsall Campus, Gorway Road, Walsall WS1 3BD, UK; ^4^Faculty of Health and Life Sciences, Coventry University, James Starley Building, Priory Street, Coventry CV1 5FB, UK

## Abstract

*Purpose*. The aims of this study were to explore the lifestyle of young people living in Al-Ahsa Governorate; to investigate differences due to gender, age, school type, and geographical location. *Methods*. 1270 volunteered youth (15–19 years) completed a self-report questionnaire that contained 47 items relating to patterns of physical activity (PA), sedentary activity, and eating habits. The questionnaire allows the calculation of total energy expenditure in metabolic equivalent (MET-min) values per week. *Results*. Significant differences in the PA levels of youth were evident with regard to gender, geographical areas, and type of school. Also, normal weight males reported the highest levels of PA compared to overweight and obese. *Conclusions*. Youth living in rural desert were less physically active than those living in urban or rural farm environments. Youth of “normal” weight were more active than obese. Males were more active than females and PA levels appeared to decline with age.

## 1. Introduction

A strong body of evidence, comprising both observational and experimental research, indicates that regular participation in physical activity (PA) among young people provides immediate and long-term benefits for physical and psychological well-being. The evidence base is growing rapidly; recent large-scale epidemiological studies utilising valid measures of PA have demonstrated stronger associations than have been observed previously and helped to clarify dose-response relationships between activity and specific health outcomes [1–3]. Physical inactivity, on the contrary, is the fourth leading risk factor for global mortality, accounting for 6% of deaths globally and ranking before overweight and obesity (5%) and after high blood pressure (13%), tobacco use (9%), and high blood glucose (6%) [4]. The benefits of regular PA have been clearly set out across the lifespan. For adults, engaging in 30 minutes of moderate intensity PA on at least 5 days a week helps to prevent and manage over 20 chronic conditions, including coronary heart disease, stroke, type 2 diabetes, cancer, obesity, mental health problems, and musculoskeletal conditions [4]. The strength of the relationship between PA and health outcomes persists throughout people's lives, highlighting the potential health gains that could be achieved if people become more active during childhood and throughout their lifespan. There is a clear relationship between the amount of PA people and all-cause mortality. While increasing the activity levels of all people who are not meeting the recommendations is important, targeting those people who are significantly inactive (i.e., engaging in less than 30 minutes of activity per week) will produce the greatest reduction in chronic disease [4].

The most recent guidelines on recommended levels of PA for children and young people (5–18 years) indicate that they should engage in moderate-to-vigorous intensity PA for at least 60 minutes and up to several hours every day, and they should minimise the amount of time being sedentary [5]. PA provides important health benefits for young people. This conclusion is based upon evidence from observational studies in which higher levels of activity were associated with more favourable health outcomes and experimental studies in which exercise treatments resulted in improvements in health-related measures [6, 7]. In addition, there is increasing evidence that the environment plays a role in influencing PA of young people and adults [8]. The physical or built environment has come to the forefront of public health research in the past 10 years, leading to a surge of research on environmental attributes and their associations with PA behaviours [9].

The Gulf Cooperation Council Countries including the Kingdom of Saudi Arabia have witnessed significant lifestyle changes due to rapid urbanisation, dominance of the automobile for personal travel, introduction of labour-saving devices in the home and the workplace, availability of high-fat and dense-caloric foods, satellite TV, increased reliance on computer, and telecommunication technology as well as decreased occupational-work demands [10]. These lifestyle changes have had a considerable impact on reducing the physical requirements of daily life and have encouraged sedentary lifestyles amongst both young people and adults. Consequently, such remarkable lifestyle transformation is thought to be greatly responsible for the epidemic of noncommunicable diseases in the whole region [11, 12]. Physical inactivity and unhealthy diets are considered among the leading causes of major noncommunicable diseases, including cardiovascular disease, type 2 diabetes, and certain types of cancer, thus contributing substantially to the global burden of disease, death, and disability in the Arab countries [13, 14]. In addition, recent research findings have shown that TV viewing (a sedentary activity) and PA appear to be separate entities and are independently associated with metabolic risk [15].

An alarming high rate of physical inactivity among the Saudi populations has been reported, predisposing them to health problems [16, 17]. Additionally, Saudi youth are not immune from the global epidemic of obesity. Although childhood obesity has been observed and widely reported in developed countries, in recent years, there is an ever-increasing prevalence in developing countries. This prevalence of childhood obesity is high in the Middle East, Central and Eastern Europe [18]. Recent national estimates of combined overweight and obesity prevalence among Saudi adolescents aged 13–18 years were 36.6% for males and 38.4% for females [19].

Studies on the physical activity patterns and sedentary behaviours as related to obesity among Saudi adolescents are scarce. Therefore, the major aims of this study were (1) to explore lifestyle and health habits of young people living in Al-Ahsa city and its surrounding area; (2) to investigate possible differences due to gender, age, school type, and geographical location. Special attention will be given to the prevalence of PA and sedentary behaviours of youth from both sexes living in rural and urban environments, and how that relates to their obesity level as assessed by Body Mass Index (BMI) and Waist Circumference (WC). It is expected that the findings from this regional study will provide substantive information on PA/inactivity patterns and health habits of young males and females in this region. Moreover, due to the limited available research data on PA and obesity measures of youth in Al-Ahsa, particularly with regard to different geographical locations, the study will afford a baseline indication of the prevalence of obesity and inactivity of youth in this region of the Kingdom of Saudi Arabia (KSA).

### 1.1. Participants

The study was carried out in Al-Ahsa Governorate located at the Eastern Province of Saudi Arabia. Since the primary objective of the study was to provide reliable population estimates of PA patterns and health habits, the sample design called for a wide geographical coverage of the Al-Ahsa schools communities (i.e., urban, rural farm, and rural desert “Bedouin”), with careful representation of the population in urban, rural, public, and private secondary schools. The sample size was determined so that it would be within ±0.05 of the population proportion with a 95% confidence level. A total of 1270 secondary-school boys and girls (15–19 years) living in Al-Ahsa Governorate volunteered to take part in this study following informed consent (participant and parental) along with institutional ethical approval. The stratified sample, representing different geographical areas of the Governorate, included 607 females (*M* = 17.07 ± 1.27 yrs) and 663 males (*M* = 17.08 ± 1.10 yrs).

### 1.2. Geographical Locations

A cross-sectional study was conducted in Al-Ahsa Governorate which is divided into five districts, namely, “Al-Hofuf, Al-Mubaraz, East villages, North villages and Al-Hejar.” A secondary school (one all boys and one all girls) from each district was randomly selected to take part in the study. Also, two private schools for each sex were randomly selected from Al-Hofuf and Al-Mubaraz districts. A total of seven schools were selected to be involved in the study representing urban, rural, and rural farm geographical locations.

### 1.3. Lifestyle Questionnaire

A validated self-report questionnaire was used to assess the PA patterns, sedentary activity, and health habits of the selected sample. This research tool was also used in the Arab Teens Lifestyle Study (ATLS) [10] and contained 47 items relating to patterns of PA, sedentary behaviours (television viewing time and video and computer use), and eating habits. The physical activity questionnaire was previously shown to have a high reliability (ICC = 0.85; 95% CL = 0.70–0.93) and an acceptable validity (*r* = 0.30; *P* < 0.05) against pedometer on a sample of males 15–25 years old [20]. In another validity study involving both males and females aged 14–19 years against pedometer, it was found to have a validity coefficient of 0.37 (*P* < 0.05) [21]. The physical activity questionnaire covers all domains of physical activity including transport, household, fitness, and sports activities. Moderate-intensity physical activity includes activities such as normal pace walking, brisk walking, recreational swimming, household activities, and moderate-intensity recreational sports such as volleyball, badminton, and table tennis. Vigorous-intensity physical activity and sports included activities such as stair climbing, jogging, running, cycling, self-defense, weight training, and vigorous-intensity sports such as soccer, basketball, handball, and singles tennis. Physical activities were assigned MET values based on the compendium of physical activity [22] and the compendium of physical activity for youth [23]. Moderate-intensity recreational sports were assigned an average MET value equivalent to 4 METs. Vigorous-intensity sports were assigned an average MET value equivalent to 8 METs. Slow walking, normal pace walking, and brisk walking were assigned MET values of 2.8, 3.5, and 4.5 METs, respectively, based on modified METs values from the compendium of physical activity for youth [24]. The questionnaire allows the calculation of total energy expenditure per week based on metabolic equivalent (MET-min) values of all types of physical activities reported by the participant. To measure the participants' levels of physical activity, the total METs min per week and the METs min per week spent in vigorous physical activity were used. The classifications adopted for activity levels in this paper were based on 2 cut-off points of 30 minutes and 60 minutes per day of at least a moderate level of physical activity. This was then converted into 3 activity categories based on total METs minute per week as follows: Active: > 1680 METs min per week (60  minutes × 7  days × 4  METs). The daily 60 minutes of at least a moderate level of physical activity is based on recent physical activity recommendations [5]. Minimally active: ≥ 840 < 1680 (30 minutes × 7 days × 4 METs). Inactive: < 840 METs min per week [6].

### 1.4. BMI Measurement

Body weight was measured to the nearest 100 grams using Seca weight scales (Seca Ltd., Hamburg, Germany). Participants were weighed barefooted and without excess outer clothing. To ensure measurement accuracy, the scale was checked for a zero reading before each weighing. Height was measured to the nearest 0.5 centimetre using a Seca portable height measure (Seca Ltd., Hamburg, Germany). BMI was calculated using the formulae: weight (kg)/height (m^2^). BMI was classified according to the International Obesity Task Force (IOTF) criteria [25].

### 1.5. Waist Circumference Measurement

Direct measurements of waist circumference were obtained to the nearest 0.5 centimetre according to Pollock and Wilmore [26]. Participants were asked to remove any outer or winter clothing (excess clothing). Waist circumference was measured horizontally to the nearest 0.5 cm using a nonstretchable measuring tape (Richter measuring tape, Seca Ltd., Hamburg, Germany) at the level of umbilical and at the end of normal expiration. The cut-off point for waist circumference classification was a waist-to-height ratio of 0.5. [27]. Those who exceed 0.5 are considered “at risk” of cardiovascular diseases. Waist circumference is considered a simple measure of fat distribution in children and adolescents and is least affected by gender, race, and overall adiposity [27, 28].

### 1.6. Statistical Analysis

A range of statistical procedures were performed on the data to establish associations and differences in the health and lifestyle habits of young people from different locations and genders. Comparisons between genders, geographical locations, and age groups were conducted using 2-way and 3-way analyses of variance (ANOVA) on the young people's PA levels, BMI, and waist circumferences. Descriptive statistics were utilised to highlight the prevalence of overweight and obesity as well as classifications according to activity index. Furthermore, Pearson's correlations were performed to establish relationships between health status variables (e.g., BMI, waist circumference, and PA levels) and sedentary lifestyle habits such as TV viewing time and computer usage. SPSS version 18 (SPSS Inc. Chicago, IL, USA) was used for all analyses.

## 2. Results

The descriptive characteristics of the main dependent variables for the total sample and subsamples are presented in Table 1. These include anthropometric, activity, and obesity measures for the participants from different geographical locations. A wide range of differences in physical activity, sedentary time, and obesity measures are evident between the two sexes and three geographical locations. 

### 2.1. Differences in Physical Activity Levels

Univariate ANOVA revealed a highly significant difference in the PA levels of young males and females in Al-Ahsa Governorate (*F*_1,1218_ = 305.61, *P* < 0.001). The differences in PA remained after controlling for adiposity (BMI and WC). The mean total METs per week for males and females was 2240.08 and 558.72 METs, respectively. Using Chi-square analysis, frequency data (i.e., Chi-square test of independence) revealed a significant difference in the PA levels between young males and females in Al-Ahsa (*χ* ^2^_2_ = 319.17, *P* < 0.001). With regard to females, there were 81.5% inactive, 14.5% minimally active, and only 4% active. Conversely, 35.7% of males were inactive, 19.8% minimally active, and 44.5% active.

**Figure 1:**
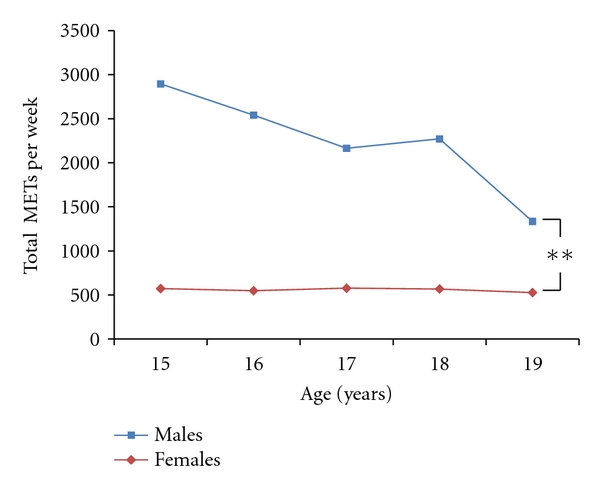
Total METs per week in males and females across different age groups. **P < 0.001 for differences in METs per week between males and females across each age group.

Univariate analyses on total PA of the whole sample revealed significant differences (*P* < 0.05) between age groups. However, this seemed to be due largely to the significant differences (*P* = 0.015) in the PA levels of males across the different age groups (Figure 1). The significant (*P* < 0.05) gender by age interaction indicates that boys' PA declined at a far greater rate than girls across the age groups. While the decline was clearly evident in males, females reported low physical activity levels across all age groups. 

**Figure 2:**
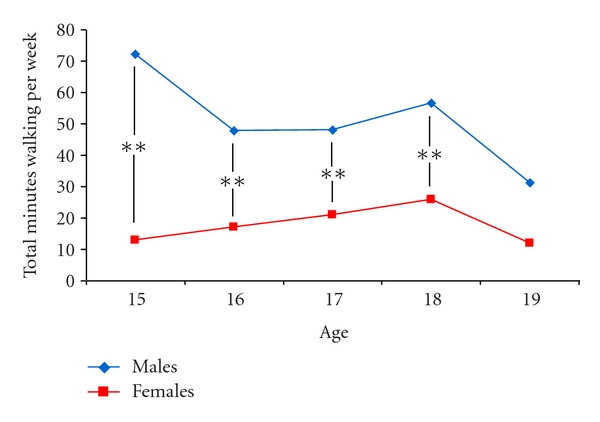
Number of minutes walking per week in males and females. **P < 0.01 for differences in the total number of minutes walking per week between males and females across each age group.

With respect to the number of minutes walking per week, the univariate analyses revealed a significant difference (*P* < 0.001) between males and females (*M* = 49.4 minutes and 19.4 minutes, resp.). However, there were no significant differences (*P* > 0.05) across the age groups (i.e., 15 to 19 years). This is illustrated in Figure 2. 

**Figure 3:**
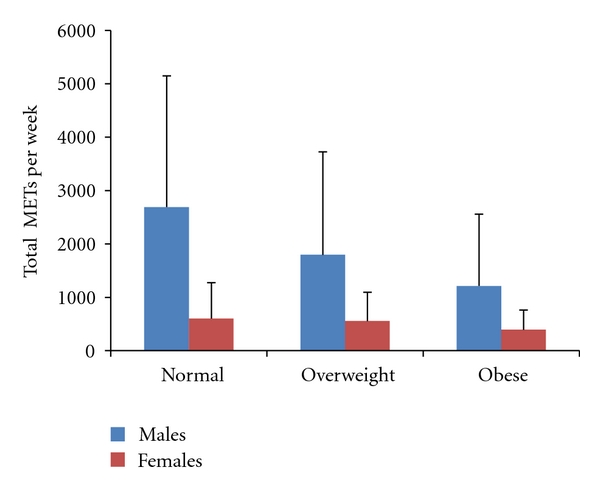
Total METs per week for males and females across obesity index.

There was also a significant main effect due to weight status in both males (*F*_2,615_ = 22.98, *P* < 0.001) and females (*F*_2,586_ = 5.03, *P* = 0.007). In both cases, young males and females of “normal” weight were the most active, while obese youth were the least active. This is highlighted by the mean values in Figure 3.

Bonferroni posthoc pairwise comparisons revealed that there were significant differences between the PA levels of normal weight, overweight, and obese males, with normal weight males reporting the highest levels of PA. However, with respect to the females, the only significant difference was between normal weight and obese youth (604 and 392 METs, resp.). 

When geographical areas of schools were considered, a highly significant difference in PA was also evident (*F*_2,1221_ = 5.95, *P* = 0.003). There was also a highly significant difference between males and females across the three geographical areas (*F*_1,1221_ = 174.21, *P* < 0.001) (see Table 1). Bonferroni posthoc analysis revealed that rural desert youth were significantly less active than those living in rural farm environments (*P* = 0.001) or urban areas (*P* = 0.023). However, there was no significant difference between males from urban and rural farm areas and between females from urban and rural desert areas. Mean (±SD) of total METs per week across gender and geographical groups is presented in Table 1. 

Univariate ANOVA revealed significant (*F*_1,1220_ = 10.422, *P* = 0.003) differences in PA levels between youth from private and public schools. However, while females in public schools were significantly more active than those in private schools (*F*_1,586_ = 7.71, *P* = 0.006), no such differences were apparent amongst males (*P* > 0.05).

### 2.2. Differences in Body Weight Classifications

Descriptive statistics indicated that both males and females exhibited a similar prevalence of overweight (16.8% and 18.8% for males and females, resp.) and obesity (19.1% and 17.7% for males and females, resp.). This equates to a total of 35.9% of males and 36.5% of females being overweight or obese. The weight status classification of youth from different geographical locations indicates that there is a high percentage of overweight and obese amongst youth of urban and rural desert. This appears to be particularly high amongst rural desert, with 51.2% of females and 43.5% of males being classified as overweight or obese (see Table 2). 

In comparing various age groups, univariate analyses revealed no significant differences in BMI (*P* > 0.05). Also, no significant differences in BMI between the schools from different geographical locations were evident. However, when examining each gender group separately, Bonferroni posthoc analysis revealed that BMI of males from rural desert was significantly (*P* = 0.004) higher than males from rural farm schools. Mean (± SD) of BMI across gender groups from different geographical environments is presented in Table 3. 

As for private and public schools, univariate ANOVA revealed no significant difference in BMI, nor was there a difference in BMI across the various age groups (both, *P* > 0.05). With respect to waist circumference, the mean values for males and females were 81.17 cm and 76.85 cm, respectively. However, with reference to the gender-specific classifications, the percentages of “at-risk” males and females were 26.5% and 54.2%, respectively (see Table 4). 

The descriptive statistics of the waist circumference for the different geographical locations indicated that the rural farm males and females had the lowest “at-risk” percentage, compared to urban and rural desert, with 34.6%, 41.5%, and 44.2%, respectively. Additionally, results indicated that 38.6% of public school males and females were “at risk” compared to 45.5% of private school (see Table 5). Also, a greater percentage of males (34.1%) in private schools were “at risk” compared to public schools (24.7%).

Pearson's correlation revealed a highly significant negative relationship between PA and BMI (*r* = −0.103, *P* < 0.001). However, when the data for males and females were analysed separately, it was evident that the relationship was only significant in the male participants. Also, there was a highly significant negative relationship between PA and waist circumference (*r* = −0.237, *P* < 0.001) even when data for each sex were analysed independently. This indicates that the more active the individual, the lower the BMI and waist circumference. 

The results revealed that there was a relationship between weight status and sedentary behaviours as represented by TV viewing, video, and computer time. For instance, in females, BMI and waist circumference were significantly related to computer time (*r* = 0.083, *P* = 0.042 and *r* = 0.121, *P* = 0.003, resp.). Moreover, in males, both BMI and waist circumference were significantly related to sedentary time (*r* = 0.110, *P* = 0.006 and *r* = 0.137, *P* = 0.001, resp.). Also, a highly significant positive relationship was evident between waist circumference and BMI (*r* = 0.766, *P* < 0.001). This supports the concurrent validity of the two measures used for weight status.

## 3. Discussion

The Kingdom of Saudi Arabia has witnessed significant lifestyle changes during the last three decades. Subsequently, physical inactivity, sedentary lifestyle, and an ever-increasing rate of obesity has become prevalent in Saudi society [29, 30]. The present study is the first of its kind to be conducted in this region to provide comparative data on the levels of physical activity of adolescents from urban and rural areas. The findings of this study show that whilst males reported more physical activity than females across the different ages (METs min per week), the significant gender by age interaction indicates that males' PA has been declining at a far greater rate than females across the age groups. This points out that the older the males were, the less active they were. The decline in PA was only clearly evident in males, with females reporting low PA levels across all age groups. This trend of declined PA with age in males and the consistently low levels of PA in females is a worrying one from a public health perspective. 

The same trend of declining PA with age was reported in other studies involving non-Saudi populations [31, 32]. Additionally, when the geographical area of the school was considered in this study, significant differences in PA between males and females were clearly evident (see Table 1). Rural desert youth were significantly less active than those living in rural farm or urban environments, possibly due to a number of environmental factors that will be explained later in this paper. As for obesity, the findings from this study indicated that both males and females exhibited a high prevalence of overweight/obesity (36.6% and 39.0%, resp.). These findings concur with a number of previous studies on Saudi youth and young adults [33–35]. The weight status classification of youth from different geographical locations indicates that there is a high percentage of overweight/obesity amongst youth from urban and rural desert areas. This appears to be particularly high amongst rural desert youth, with 51.2% of females and 43.5% of males being classified as overweight or obese (see Table 2). With respect to waist circumference, the mean values for males and females were 81.17 cm and 76.85 cm, correspondingly. As for the gender-specific classifications, the percentage of “at-risk” males and females were 26.5% and 54.2% (see Table 4), signifying that there was a seriously high percentage of “at-risk” youth, particularly amongst females.

There are a number of plausible explanations for the findings of this study with regard to rural desert youth's PA and obesity. The low levels of PA and higher percentage of obesity amongst rural desert youth compared to those living in rural farm or urban environments might be due to a number of environmental factors. Firstly, with regard to PA, the harsh desert climate, which is extremely hot in summer and very cold and windy in winter, is normally not conducive to engagement in PA for a substantial part of the year, a problem further compounded by the absence of appropriate facilities for exercise in these locations. This might be part of the reason why youth in rural desert areas are less active than youth in rural farm areas. On the other hand, youth in rural farm lands are expected to take part in some of the farming activities that are physically demanding, like ploughing, planting, and harvesting. In general, engagement in PA by young people, particularly in rural environments of Saudi Arabia, is not regarded as a desired pursuit (leisure time activity) due to cultural attitudes and beliefs. It is usually perceived that the pursuit of academic excellence has greater status than PA. Normally, parents encourage their children to engage in educational and spiritual activities rather than leisure time physical activities. Additionally, there is a general lack of availability of parks, sports grounds, and facilities that are suitable for youth to get involved in PA or sports. Moreover, attitudes, societal norms, and expectations of rural desert communities are less encouraging towards engagement in sporting activities that require adherence to particular outfits/clothing than other communities. Moreover, youth living in rural farm or urban environments, normally, have better access to sports facilities compared to rural desert youth. The noticeable and consistent difference in PA of males and females across the age groups in this region could be due to a number of cultural and environmental factors; these may include social norms, expectations, and perceptions as well as the opportunities for PA afforded by both the home and the wider community for males and females. 

Secondly, with regard to weight classification, the current study revealed that the combined prevalence of overweight and obesity was 37.78% with 19.63% overweight and 18.15% obese amongst the 1270 participants. A previous study on adolescents from Al-Ahsa region by Amin et al. [33] revealed a combined prevalence of obesity and overweight of 23.9% (9.7% obese and 14.2% overweight) among males aged 10–14 years. Hence, the findings of the current study draw attention to the fact that the prevalence of obesity amongst youth in this region seems to be on the increase in recent years. Also, an earlier cross-sectional household survey that involved 13,177 youth and adult Saudis aged 15 years and over, indicated that the prevalence of overweight (BMI 25–30) was higher among males than females (29% versus 27%), while the prevalence of obesity (BMI >30) was higher among females than males (24% versus 16%) [34, 35]. This equates to 45% of males and 51% of females being classified as overweight or obese. Furthermore, an earlier study by El Mouzan et al. [19] reported that the overall prevalence of overweight was 11.7% and obesity 15.8% amongst males aged 6 to 18 years. The highest prevalence of obesity was recorded in the capital city of Riyadh (18%). A more recent study on the dietary behaviour and lifestyle of Saudi female university students reported an overweight of 31.4% and obesity of 16.5%. This represents a total of 47.9% of young female adults who were either overweight or obese [36]. Additionally, a recent systematic review paper on obesity in Gulf Co-operation Council States [37] that reviewed 45 studies, reported prevalence of overweight and obesity in adults of 25–50% and 13–50%, respectively, with a higher prevalence of obesity amongst women. The findings of these studies point to the increasing prevalence of obesity in recent years.

Moreover, the findings of the current study revealed that the prevalence of obesity amongst Saudi youth has matched or exceeded that of other regions of the world. For example, Wang and Lobstein [38] reported a combined prevalence of overweight and obesity of 46.4% in the Americas, 41.7% in Eastern Med, 38.2% in Europe, and 22.9% in South East Asia. This signifies the urgency of formulating policies and designing intervention strategies to reverse and reduce the epidemic of obesity and safeguard the future health of young people and adults in Saudi Arabia. The findings have also indicated that the young males of “normal” weight were the most active, while the obese were the least active; this is illustrated by the mean values in Figure 3. Whilst “normal” weight males were significantly more active than both overweight and obese males, in females, those with “normal” weight were only significantly more active than the obese females. The descriptive statistics with respect to waist circumference revealed that the youth from rural farm locations had the lowest percentage of “at risk” compared to youth from urban and rural desert locations, with 34.6%, 41.5%, and 44.2%, respectively. It is noteworthy to point out that these results seem to reflect the level of PA across the geographical locations, with males and females from rural farm locations being the most physically active and the least “at-risk” group. Additionally, the rural desert youth appeared to be the least active and had the highest prevalence of overweight/obesity (47.3%) compared to both the urban (38.3%) and rural farm (27.6%) youth. Recent studies on populations from other countries have pointed out differences between urban and rural youth. A large national cross-sectional population survey of 5–15 year old New Zealanders [39] found that rural children had a significantly lower BMI, smaller waist circumferences, and thinner skinfold measurements than urban children. There was no significant difference in the energy intake per day of rural and urban children. Similarly, there was no significant difference in the frequency of bouts of physical activity undertaken by rural and urban children. Also, in another study involving 362 Portuguese youth (165 males, 197 females) 13–16 years of age representing urban or rural residence, youth of both sexes from rural settings were 76% more likely to be classified as aerobically fit compared to those from urban areas [40]. This study suggested that the interaction of several environmental factors such as age, gender, weight status, parental education, and screen time may explain why rural Portuguese youth were more likely to be classified as physically fit compared to urban youth. 

The low levels of PA and high percentage of overweight/obese amongst males and females in the current study might be related to certain aspects of their lifestyle, dietary habits, and environmental factors. For example, using saturated fat in traditional cooking is commonplace in this region. A recent study by Washi and Ageib [41] on poor diet quality and food habits of Saudi youth found an increase in dietary intake or energy from fats as well as the fact that rice, bread, and meat are regarded as the staple diet, which are used in almost every meal. This seems to concur with other studies (dealing with this age group) which found that obese children and adolescents consume significantly more servings of meat, grain products, fast foods, sugar, sweetened drinks, and potato chips. These contribute to a higher caloric intake compared to nonobese children and adolescents [42]. 

Research findings have pointed out that the prevalence of obesity amongst youth over the past three decades seems to be increasing in almost all industrialized countries and in several lower-income countries [38]. In societies that have been undergoing rapid socioeconomic transitions (e.g., Saudi Arabia), obesity has increased at an accelerated rate with the prevalence of overweight or obesity in school-age children doubled or tripled in several industrialized countries, such as Canada, the United States, Brazil, Greece, and the UK [37]. A strong inverse relationship between socioeconomic status and obesity appears to exist among women in developed countries, but this relationship is inconsistent for men and children. In contrast, in developing countries a strong relationship exists between socioeconomic status and obesity among men, women, and children [43]. In the current study, overweight and obesity were associated with different geographical locations and socioeconomic backgrounds as represented by type of school attended: private or public. A higher percentage of overweight and obese youth was found amongst those who live in urban and rural desert areas. This appears to be particularly high amongst the rural desert youth, with females and males having 51.2% and 43.5% in the overweight/obese category respectively. Additionally, this study found that there was a relationship between levels of PA and obesity, with those reporting higher levels of PA tending to possess lower BMI. However, this relationship was only significant amongst male participants. A similar relationship was evident between PA and waist circumference. This indicates that the more active the individual, the lower the BMI and waist circumference. Furthermore, a relationship was evident between weight status and sedentary lifestyle, with a higher BMI recorded by youth reporting greater time use of computer. Moreover, a larger waist circumference was recorded by youth who reported more sedentary time (TV viewing and computer time).

The current study indicated that females in public schools were significantly more active than those in private schools. This might be due to socioeconomic differences such as the excessive use of automobile transportation, availability of satellite TV, computers, and other electronic devices that encourage inactivity. The combined impact of high-fat diets, reduced PA, and the traditional societal perception of fatness as a sign of affluence and beauty could be significant contributors to the prevalence of obesity amongst more affluent females. Socioeconomic differentials may be associated with the problem of overweight and obesity amongst youth in Al-Ahsa. This is reflected by the high number of “at-risk” youth amongst both males and females in private schools. Moreover, since the prevalence of sedentary lifestyle and obesity seems to increase with age, one would expect this problem to get even more serious in the future, as the participants get older. 

There are a number of limitations to this study. In addition to the inherent limitations of self-report approaches to the assessment of PA and sedentary behaviour, the instrument used here may have been less sensitive to the normal daily PA of youth in rural desert. Additionally, the differences in maturation status between youth from the three geographical groups may have affected obesity classification. Further studies on lifestyle and health habits of youth in the Al-Ahsa Governorate are needed. These may involve the use of objective methods of assessing PA and investigate other risk factors of the metabolic syndrome.

## 4. Conclusions

This study has a number of strengths, including being the first study in the region to provide comparative data on PA and obesity of youth from urban and rural areas. The study used an appropriate tool for data collection and recruited a representative sample of the geographical areas of the region under study to explore environmental differences. The findings demonstrated that factors such as gender, age, and geographical location seem to influence youth PA levels and obesity. Youth living in rural desert were less physically active than their counterparts living in urban or rural farm environments. Young males of “normal” weight were more active than their overweight and obese counterparts. Also, PA levels of young males, as measured by METs min per week, appear to decline gradually with age, indicating that the older the males, the less active they were. Moreover, males were generally more active than females, with females exhibiting higher rates of obesity and acutely lower levels of PA across all ages.

## Figures and Tables

**Table 1:** Mean ± SD of the main dependent variables for the total sample and sub-samples.

Variable	Urban		Rural farm		Rural desert		Whole group	
	Male *n* = 371	Female *n* = 333	Male *n* = 207	Female *n* = 189	Male *n* = 85	Female *n* = 85	Male *n* = 663	Female *n* = 607
Age	17.03 ± 1.02	16.78 ± 1.21	17.13 ± 1.25	17.50 ± 1.21	17.15 ± 1.04	17.24 ± 1.35	17.08 ± 1.10	17.07 ± 1.27
Weight	70.08 ± 21.62	60.47 ± 20.03	63.31 ± 18.37	54.12 ± 15.51	69.10 ± 20.05	62.23 ± 18.83	67.76 ± 20.62	58.74 ± 18.80
Height	168.6 ± 7.41	154.91 ± 9.00	166.86 ± 6.61	153.75 ± 8.02	167.29 ± 6.07	154.16 ± 5.74	167.86 ± 7.02	154.44 ± 8.32
BMI	24.58 ± 7.18	26.35 ± 21.84	22.67 ± 6.16	23.89 ± 20	24.59 ± 6.66	26.00 ± 6.84	23.97 ± 5.764	25.54 ± 19.83
Abdominal Circumference	78.73 ± 16.87	82.47 ± 15.02	73.90 ± 14.15	78.58 ± 12.65	76.36 ± 13.91	81.89 ± 15.24	76.85 ± 15.79	81.17 ± 14.44
Time spent on TV	2.60 ± 1.81	2.28 ± 1.67	2.29 ± 1.62	2.71 ± 1.97	2.48 ± 2.13	3.63 ± 2.20	2.49 ± 1.80	2.60 ± 1.90
Time spent on computer	2.58 ± 2.02	3.57 ± 2.45	2.34 ± 2.05	2.98 ± 2.29	1.96 ± 2.26	2.12 ± 2.09	2.43 ± 2.07	3.19 ± 2.40
Total METs min/week (vigorous)	1780 ± 2037	112 ± 76	1653 ± 2066	176 ± 410	1286 ± 1448	64 ± 41	1678 ± 1987	125 ± 232
Total METs min/week (moderate)	702 ± 772	475 ± 409	740 ± 841	696 ± 584	368 ± 358	540 ± 394	676 ± 769	564 ± 490

**Table 2:** BMI classifications according to IOTF.

		BMI classification		
Geographical location		Normal *n* = 770	Overweight *n* = 211	Obese *n* = 220
Urban	Males	60.5%	17.6%	21.9%
	Females	62.8%	16.8%	20.4%
	Both sexes	61.6%	17.2%	21.2%

Rural farm	Males	73.3%	12.6%	14.1%
	Females	71.4%	17.5%	11.1%
	Both sexes	72.4%	14.9%	12.7%

Rural desert	Males	56.5%	23.5%	20%
	Females	48.8%	29.8%	21.4%
	Both sexes	52.7%	26.6%	20.7%

All	Males	64.1%	16.8%	19.1%
	Females	63.5%	18.8%	17.7%

**Table 3:** BMI according to geographical location of schools.

	Urban *n* = 684	Rural farm *n* = 385	Rural desert *n* = 152
Males	24.58 ± 7.18	22.67 ± 6.16	24.59 ± 6.66
Females	26.35 ± 21.84	23.89 ± 19.20	26.00 ± 6.84

**Table 4:** Waist circumference classification according to gender and geographical location.

Subgroups	Waist circumference classification							
	Urban		Rural farm		Rural desert		Total sample	
	Normal *n* = 383	“At risk” *n* = 272	Normal *n* = 248	“At risk” *n* = 131	“Normal” *n* = 92	“At risk” *n* = 73	Normal *n* = 723	“At risk” *n* = 476
Males	75.3%	24.7%	79.7%	20.3%	70.2%	29.8%	73.5%	26.5%
Females	49.5%	50.5%	49.2%	50.8%	40.7%	59.3%	45.8%	54.2%
Whole group	58.5%	41.5%	65.4%	34.6%	55.8%	44.2%	60.3%	39.7%

**Table 5:** Waist circumference classification according to school type and gender.

Subgroups	Waist circumference classification			
	Public		Private	
	Normal *n* = 231	“At risk” *n* = 145	Normal *n* = 152	“At risk” *n* = 127
Males	75.3%	24.7%	65.9%	34.1%
Females	49.5%	50.5%	37.5%	62.5%
Whole group	61.4%	38.6%	54.5%	45.5%
